# If at First You Don't Succeed, Try, Try Again? Antipsychotic Trials and Clozapine Provision in Glasgow's Esteem (Early Intervention in Psychosis) Service

**DOI:** 10.1192/bjo.2024.543

**Published:** 2024-08-01

**Authors:** Matthew Beattie, Josie Nott, Rajeev Krishnadas

**Affiliations:** 1 NHS Greater Glasgow & Clyde, Glasgow, United Kingdom

## Abstract

**Aims:**

To support evidence gathering for Esteem's RCPsych Early Intervention in Psychosis (EIP) network accreditation efforts, an audit was conducted to investigate compliance with EIPN's quality standards (QS) no. 33 and no. 36.

EIPN QS 33 = patients with first episode psychosis (FEP) are offered antipsychotic medication.

EIPN QS 36 = If the patient's illness does not respond to an adequate trial of two different antipsychotic medicines given sequentially, they are offered clozapine.

EIPN QS 36 is also specifically included in RCPsych's National Clinical Audit of Psychosis (NCAP) (listed as standard 4), but a more pragmatic definition is used, to factor in the issue of antipsychotic intolerance.

NCAP Standard 4 = People with FEP who have not responded adequately to or tolerated treatment with at least two antipsychotic drugs should be offered clozapine (NICE QS80).

This broader standard definition was used for this audit, to allow for results comparison with national data.

**Methods:**

For EIPN QS 33, all patients on North East Esteem caseload (any primary diagnoses) for at least 6 months on 01/04/2023 were included.

For EIPN QS 36/NCAP Standard 4, the same inclusion criteria were used but refined to FEP cases only.

The electronic clinical records (EMIS) of such cases were reviewed manually by an ST5 and CT3 psychiatrist. Data on prescription history was collected then analysed in Microsoft Excel.

**Results:**

EIPN QS 33: 58 patients with any primary diagnosis were initially identified as being on NE Esteem caseload > 6 months as of 01/04/23. 58 (100%) patients were offered antipsychotic medication ⋅ 1 (2%) patient was prescribed an antipsychotic but never took it ⋅ 21 (36%) patients were only ever prescribed one antipsychotic ⋅ 17 (29%) patients were prescribed two antipsychotics sequentially trialled ⋅ 11 (19%) patients were prescribed three antipsychotics sequentially trialled ⋅ The remainder, 8 (14%) patients, had four or more antipsychotics sequentially prescribed (with the maximum number of trials being eight).

EIPN QS 36 / NCAP Standard 4: 55 patients with FEP diagnosis were initially identified as being on NE Esteem caseload > 6 months as of 01/04/23. 16 (29%) of these patients had at least three or more trials of antipsychotic medication, i.e. patients eligible for clozapine. However, only 5 (31%) of these 16 patients had either been prescribed clozapine (3 patients, 19%) or offered/trialled clozapine (2 patients, 13%). This 31% figure compares with 85% in Wales, 52% in England, and 50% in Ireland (NCAP 2021–22).

**Conclusion:**

EIPN QS 33: The standard that patients with first episode psychosis are offered antipsychotic medication was fully met. About a third of patients required only one antipsychotic trial. Less than a third required two antipsychotic trials. One in five required three antipsychotic trials, and approximately one in seven patients required more than three antipsychotic trials.

EIPN QS 36/NCAP Standard 4: The number of eligible patients being offered or prescribed clozapine for first episode psychosis under care of NE Esteem falls well below NCAP averages for Wales, England and Ireland.

